# Colloid Degeneration of the Omentum

**Published:** 1880-01

**Authors:** A. G. Crittenden

**Affiliations:** Clifton Springs, N. Y.


### COLLOID DEGENERATION OF THE OMENTUM.

REPORTED BY A. G. CRITTENDEN, M. D., CLIFTON SPRINGS, N. Y.

Mr. B., aged sixty-three years, temperate, of active habits, light complexion, died in May last. Three years previously he complained of some renal disease, acidity of the stomach, abdominal enlargement, and at times nausea and vomiting; was tapped seven times, the fluid being thin at first, and afterwards thick as jelly. During all this time there was but little abdominal tenderness and pain.

Autopsy twenty hours after death: The omentum weighed fifteen pounds, and was composed of fibrous tissue, the interstices being filled with white jelly like bodies the size of peas and smaller; a mass of colloid, in quantity about two quarts, of the consistency of the white of egg, apparently secreted from the omentum and peritoneal coating of the intestines; portions of the peritoneum were ulcerated in circular patches; between the peritoneum and skin, in the abdominal walls, were found several round bodies as clear as glass, sections of which were of the size of a dime, and smaller; other organs were nearly healthy.

